# The Physiological and Biochemical Responses of *Daphnia magna* to Dewatered Drinking Water Treatment Residue

**DOI:** 10.3390/ijerph17165863

**Published:** 2020-08-13

**Authors:** Nannan Yuan, Yuansheng Pei, Anping Bao, Changhui Wang

**Affiliations:** 1 Electronic Information Technology School, Nanjing Vocational College of Information Technology, Nanjing 210023, China; yuannn@njcit.cn (N.Y.); baoap@njcit.cn (A.B.); 2 State Key Laboratory of Lake Science and Environment, Nanjing Institute of Geography and Limnology, Chinese Academy of Sciences, Nanjing 210008, China; 3 State Key Laboratory of Water Environment Simulation, Key Laboratory for Water and Sediment Sciences of Ministry of Education, School of Environment, Beijing Normal University, Beijing 100875, China; yspei@bnu.edu.cn

**Keywords:** environmental remediation, sediment, heavy metals

## Abstract

There have been widespread attempts to recycle drinking water treatment residue (DWTR) after dewatering for environmental remediation, which is beneficial for both the environment and the economy. The directly discharged DWTR without dewatering to natural water bodies, however, was reported to show signs of chronic toxicity to *Daphnia magna* (*D. magna*), a typical zooplankton in the aquatic environment. This study comprehensively assessed the effect of dewatered DWTR on the physiological and biochemical characteristics of *D. magna* based on acute and chronic toxicity tests. The results showed that the survival, growth, reproduction, body morphology of offspring, and the antioxidant enzymes of *D. magna* were not affected by the dewatered DWTR. These physiological and biochemical indexes also had no undesirable changes for the DWTR-amended sediments (with ratios of 0–50%) incubated for 10 and 180 d; the growth and reproduction were even promoted when *D. magna* was exposed to 5000 mg-sediment L^−1^, which may be due to the extra nutrients supplied by the amended sediments for the animals. The results demonstrated that by contrast with the directly discharged DWTR without dewatering, the dewatered DWTR could be safe to *D. magna*. Further analysis suggested that heavy metals (Pb, Ni, Cu, Cr, and Zn) with relatively low concentrations and high stability could be the main reasons leading to the high safety of the dewatered DWTR. Overall, dewatered DWTR can be considered a non-hazardous material for zooplankton.

## 1. Introduction

Drinking water treatment residue (DWTR) is an inevitable byproduct generated largely during potable water production [1]. Commonly, the by-product is disposed of in two different ways: one is disposal into landfills following dewatering, namely dewatered DWTR; the other is direct discharge into surface water or urban sewage systems without dewatering, namely drinking water treatment sludge (DWTS) [2]. Due to the increased demand for potable water for the growing human population, the production of DWTR tends to increase, leading to higher economic and environmental costs for disposal [2]. Accordingly, DWTR (mainly referring to the dewatered DWTR) recycling was of particular interest to scientists in the past decades [3,4].

DWTR is typically considered to be a potential resource for environmental remediation [1,4], as it has been shown to have high adsorption capability for various pollutants, such as antibiotics [5], fluoride [6], perchloric acid [7], sulfide [8], metals (metalloids) [9], and phosphorus (P) [10]. As a consequence, DWTR has been reused to remediate soils contaminated by metals [11], to reduce the excessive loss of P from P-rich soil [12], to remove excessive P from wastewater as the main substrate for constructed wetlands [13], and to control sediment pollution of P and metals [14,15]. Such efforts undoubtedly can promote DWTR recycling.

Assessment of the environmental safety is another important support for DWTR recycling in environmental remediation. Because DWTR mainly consists of inorganic components [2], the risks of heavy metal pollution have been investigated widely. The findings have indicated that most of heavy metals in DWTR tend to be at low concentrations and have high stability under natural conditions (e.g., pH 6–9 and various redox conditions) [16,17,18,19]. The relatively low concentrations of organic pollutants also have been reported in DWTR, although careful management is recommended for DWTR in cyanobacteria bloom-affected areas [20].

However, the reports about the DWTR ecotoxicity exhibit certain differences. The dewatered DWTR shows no toxic effect on the alga *Chlorella vulgaris* (*C. vulgaris*) and the bacterium *Aliivibriofischeri* (*A.fischeri*) [21,22,23]. In contrast, the directly discharged DWTS in natural water bodies tends to reduce the fecundity of *Daphnia magna* (*D. magna*), showing signs of chronic toxicity to *D. magna* [24,25]. The heavy metals (zinc (Zn), lead (Pb), nickel (Ni), copper (Cu), and chromium (Cr)) were identified as the main causes underlying chronic DWTS toxicity [24,25]. The different reports about the potential toxic effect between the dewatered DWTR and the discharged DWTS indicated the potential requirement for assessing the toxicity of the dewatered DWTR to *D. magna* to address concerns about the safety of recycling DWTR in environmental remediation.

Therefore, the effects of dewatered DWTR on the survival, growth, life-history characteristics, body morphology of offspring, and antioxidant enzyme activities of *D. magna* were examined in this study. Because DWTR was typically recycled in natural water remediation [1,4], the bioassay was performed based on both DWTR and DWTR amended lake sediments. The results of this study will facilitate the productive reuse of dewatered DWTR during environmental remediation.

## 2. Materials and Methods

### 2.1. Sample Collection and Preparation

Dewatered DWTR was collected from a Beijing drinking water plant. Fresh dewatered DWTR was air-dried, broken, and sieved through a diameter of less than 1 mm. Sediment was obtained from Lake Baiyangdian (38°53′ N, 115°59′ E) in north China. The upper 10 cm of the sediment was collected, filtered through a 1.8 mm screen to remove impurities, and then homogenized mechanically. The sediment was stored at 4 °C until use (within 48 h).

The preparations of the DWTR amended sediment are described below. DWTR was completely mixed with sediment at doses respectively accounting for 0, 10, and 50% of sediment in dry weight, and the mixtures were then incubated in the dark. On day 10 and day 180, the mixtures were sampled, freeze-dried, broken, and sieved to a diameter of less than 1 mm. The samples were named SNR-10 for sediments without DWTR incubated for 10 days, SR1-10 for sediments with 10% DWTR incubated for 10 days, SR5-10 for sediments with 50% DWTR incubated for 10 days, SNR-180 for sediments without DWTR incubated for 180 days, SR1-180 for sediments with 10% DWTR incubated for 180 days, and SR5-180 for sediments with 50% DWTR incubated for 180 days.

### 2.2. Physico-Chemical Properties Analysis

The total contents of iron (Fe), aluminum (Al), Zn, Pb, Ni, Cu, and Cr in samples were determined according to the Method 3051 [26]. The metals lability in samples was assessed by the European Community Bureau USEPA of Reference (BCR) sequential extraction procedure, which separated the extracted metal into three fractions, i.e., acid-soluble, reducible, and oxidizable metals, respectively [27]. The extracted metals were analyzed by inductively coupled plasma-atomic emission spectrometry or inductively coupled plasma mass spectrometry (Agilent 7700x, Agilent, CA, USA). The pH was measured in the supernatant of a 1:2.5 suspension (solid:solution; g mL^−1^) prepared by CO_2_-free water (pH-10, Sartorius, Germany). Total organic matter was determined based on dichromate oxidation method [28]. The particle size distributions (Mastersizer 2000, Mastersizer, Malvern, UK) were also determined. Each test was run in triplicate.

### 2.3. Test Organism

The test organism, *D. magna*, was obtained from the Chinese Center for Disease Control and Prevention (Beijing, China), which have been cultured in laboratory conditions for over 10 years. The culture was maintained in synthetic freshwater (OECD standard test medium, STM) (OECD 202) to ensure that *D. magna* could survive at least 2 days without food supplementation and in a temperature-controlled incubator at 25 ± 0.5 °C with a 16–8 h light-dark photoperiod. *Daphnia magna* were fed daily with the green algae *Scenedesmus obliquus*, and culture medium was renewed three times a week. In addition, tests with the reference chemical K_2_Cr_2_O_7_ were run in 24 h to evaluate the sensitivity of *D. magna* (ISO6341:2012) [29].

### 2.4. Biological Tests

Four sets of tests were performed for DWTR, SNR-10, SR1-10, SR5-10, SNR-180, SR1-180, and SR5-180 (Table 1). Neonates less than 24 h in age were used for the first three sets of experiments (i.e., bioassay 1–3), and *D. magna* at least 5 days in age were used for the last sets of tests (i.e., bioassay 4). Bioassay 1, 3, and 4 were conducted under the same temperature and light conditions as described in Section 2.3*,* while bioassay 2 was conducted at 25 ± 0.5 °C in darkness.

Bioassay 1 was conducted under static and non-renewal conditions for 48 h to determine the mortality of *D. magna* in 50 mL glass beakers with 20 mL of test media. Samples were tested at 0, 5, 50, 100, 500, and 5000 mg L^−1^. Five replicates with five *D. magna* in each repeat were conducted. Before the test, the organisms were fed *Scenedesmus* for 2 h (ISO6341:2012) [29,30].

Bioassay 2 was performed to determine the growth of *D. magna* with 100 mL of test media at sample concentrations of 0, 5, 50, 100, 500, and 5000 mg L^−1^. Five replicates with five *D. magna* in each repeat were conducted. Neonates in each beaker were fed with *Scenedesmus* at a concentration of 5 mg-C L^−1^. Growth rates were determined as the increase in dry mass and body length from the beginning of the experiment (W_0_ and L_0_) to day 5 (W_5_ and L_5_) using the following equations [31]:

(1) $$\mathrm{Body}\;\mathrm{weight}\;\mathrm{growth}\;\mathrm{rate}=({\mathrm{lnW}}_{5}-{\mathrm{lnW}}_{0})/5,$$

(2) $$\mathrm{Body}\;\mathrm{length}\;\mathrm{growth}\;\mathrm{rate}=({\mathrm{lnL}}_{5}-{\mathrm{lnL}}_{0})/5,$$

Bioassay 3 was conducted to examine the responses of life-history traits of *D. magna* at sample concentrations of 0, 500, and 5000 mg L^−1^. *Scenedesmus* was added daily to all beakers as food. Ten replicates with only one *D. magna* in each repeat were conducted. The numbers of survivors, time to first pregnancy, and time to release the first brood were recorded, and newborns were removed and counted to obtain the average number of neonates produced by each organism during the 21-day test period. At the end of the exposure period, the body lengths of animals were measured. Offspring born during the first brood of test organisms was transferred to and cultured in fresh test medium for 9 days. Next, offspring were inspected and photographed under a binocular microscope and classified either as normally or abnormally developed [32].

Bioassay 4 was conducted to analyze the antioxidant enzymes of *D. magna* after 48 h of exposure to samples at concentrations of 0, 500, and 5000 mg L^−1^ [33]. Five replicates of five *D. magna* each were conducted. The tests were performed under static non-renewal conditions in 50 mL of test solution without feeding. After the tests, the activities of catalase (CAT), superoxide dismutase (SOD), glutathione peroxidase (GPX), and glutathione-S-transferase (GST) were determined using the reagent kits (Nanjing Jiancheng Bioengineering Institute) per the manufacturer’s instructions. The detailed methods are shown in the Supporting Information.

### 2.5. Statistical Analysis

Any significant differences between the control and experimental treatments were determined using analysis of variance (ANOVA) followed by the least significant difference (LSD) test. Statistical significance was accepted at *p* < 0.05. Error bars represented the means ± standard deviations of repeat measurements.

## 3. Results

### 3.1. The Properties of Drinking Water Treatment Residue (DWTR) and Sediment

The properties of DWTR and sediments are listed in Table 2. The pH of DWTR, raw sediment, and DWTR amended sediments were similar and within 7.30–7.90, while other properties had some differences. Compared to raw sediment, DWTR contained higher contents of organic matter, Fe, Al, Pb, Ni, Cu, and Cr, with lower contents of Zn. The total heavy metal contents (Pb, Ni, Cu, Cr, and Zn) between DWTR and raw sediment were mainly in the same order of magnitude, which led to the limited variations of these metals contents in sediment after being amended by DWTR at different dosages (0–50%) and incubation time (10 and 180 d). Nevertheless, DWTR addition tended to increase total Al and Fe content in sediment with different incubation time. In addition, 27.3–41.4% of solid particles in sediments with and without DWTR addition had diameters less than 40 μm, but the value was only 2.45% for DWTR.

The results of metals fractionation suggested that metals in raw sediments were mainly in residual fractions (80.6–97.3%), except that Pb was dominated by oxidizable (38.5%) and residual (60.4%) fractions. In DWTR, distributions of Cr and Pb were similar to those in raw sediments; Fe, Al, Pb, and Cu were mainly in oxidizable (48.2–58.7%) and residual (27.5–49.1%) fractions; Ni and Zn were generally distributed in all four fractions. The distributions of metals were similar between raw sediment and the incubated sediments without DWTR addition (at both 10 and 180 d). Further analysis showed that the addition of DWTR led to the transformation of Al, Fe, Zn, and Cu from residual to oxidizable fractions, of Pb from oxidizable to residual fractions, with limited effect on the distributions of Cr and Ni in sediments. As DWTR doses increased, the enhanced transformation was observed for Al, Fe, Zn, and Cu in sediments; the distributions were similar to each amended sediment between 10 and 180 d incubation. These results suggested that the distributions of metals varied in DWTR and sediment, and that different metals had different concentrations in sediment after being amended with DWTR.

### 3.2. The Survival of D. magna

The acute toxicity result of K_2_Cr_2_O_7_ to *D. magna* is shown in Figure S1. In the control group, the percentage of dead *D. magna* was less than 10% after the experiment, indicating that the results of the tests were reliable and effective. The EC_50_ value for *D. magna* exposed to K_2_Cr_2_O_7_ was 1.70 mg L^−1^ within the confidence interval of 1.53 to 2.00 mg L^−1^. The result suggested that the sensitivity of *D. magna* used herein met the specified requirements from ISO6341:2012, which showed that the EC_50_ should be 0.9–1.7 mg L^−1^.

The survival of *D. magna* is shown in Table 3. In the control group, 96% of *D. magna* survived. 100% of *D. magna* survived in most of the experimental treatments (with DWTR and sediments), except for survival of 96% in the 100 mg L^−1^ DWTR group, 98% in 5 mg L^−1^ SNR-10 group, 96% in 50 mg L^−1^ SR1-10 group, and 98% in the 100 mg L^−1^ SNR-180 group. The relatively high survival of *D. magna* in the experimental treatments (compared to the control) suggested that dewatered DWTR and DWTR-amended sediments did not cause acute toxicity to *D. magna*.

### 3.3. The Growth of D. Magna

**Figure 1:**
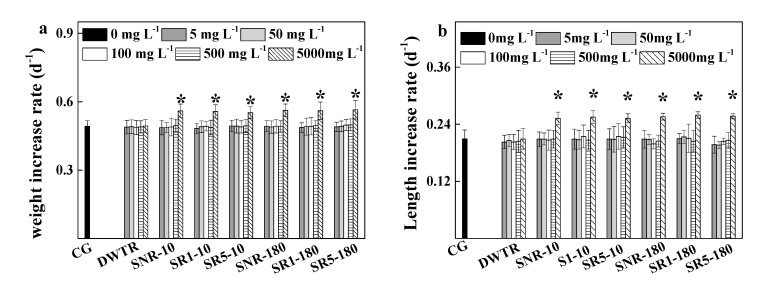
Weight increase rate (**a**) and body length increase rate (**b**) of *D. magna*. * Above the columns represents statistical significance (*p* < 0.05) between results of the control and the treatments.

The growth of *D. magna* is shown in Figure 1. During the tests, all animals survived in the controls and experimental treatments (with DWTR and sediments). Based on the increases in body weight and length over time, growth rates of *D. magna* were generally similar between the control and the experimental treatments, as well as between the treatments of sediments with and without DWTR addition (at the incubation time of 10 and 180 d with 10% and 50% doses), except that in the treatments of 5000 mg L^−1^ sediments (amended with and without DWTR), growth rates were significantly higher (*p* < 0.05) than in the control. Specifically, the growth rates calculated based on the body weight and length of the juveniles were 0.492 (±0.024) and 0.209 (±0.018) d^−1^ in the control; while the rates were respectively 0.552 (±0.026)–0.564 (±0.040) and 0.252 (±0.010)–0.259 (±0.007) d^−1^ for the treatments of different sediments at a concentration of 5000 mg L^−1^ sediments. The results demonstrated that DWTR and DWTR-amended sediments did not inhibit the growth of *D. magna*, and the growth tended to be promoted by sediments with and without DWTR.

### 3.4. The Life-History Traits of D. magna

**Figure 2:**
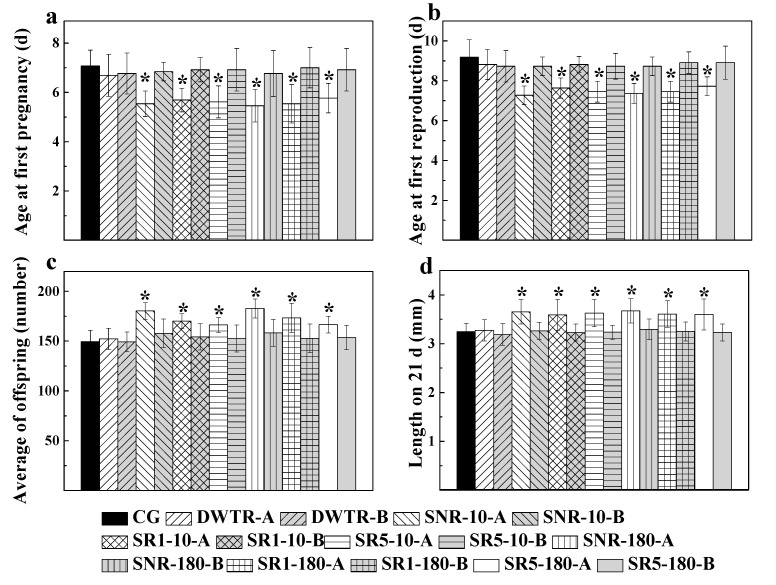
Age at first pregnancy (**a**), age at first reproduction (**b**), average number of offspring (**c**), and length on 21 d (**d**) of *D. magna*. A and B represent the concentrations of treatment culture at 5000 and 500 mg L^−1^, respectively; CG represents control group; * above the columns represents statistical significance (*p* < 0.05) between results of the control and the treatments.

The life-history traits of *D. magna* are shown in Figure 2. The age at first pregnancy, age at first reproduction, average number of offspring, and length at 21 d in age were generally similar between the control and the experimental treatments (with DWTR and sediments), as well as between the treatments of sediments amended with and without DWTR (at the incubation time of 10 and 180 d with 10% and 50% doses), except that significant differences (*p* < 0.05) were observed between the control and the treatments of 5000 mg L^−1^ sediments (with and without DWTR). Specifically, age at first pregnancy, age at first reproduction, average number of offspring, and length at 21 d were, respectively, 7.08 (±0.64) d, 9.18 (±0.87) d, 150 (±11), and 3.25 (±0.17) mm in the control. For 5000 mg L^−1^ sediments (with and without DWTR), first pregnancy and reproduction began earlier at 5.46 (±0.66)–5.77 (±0.60) and 7.27 (±0.47)–7.73 (±0.47) d, respectively, and average number of offspring and length at 21 d increased to 166 (±8)–182 (±10) and 3.59 (±0.31)–3.67 (±0.25) mm, respectively. These results indicated that DWTR and DWTR-amended sediments had no adverse effect on the life-history characteristics of *D. magna*, and sediments with and without DWTR addition at a concentration of 5000 mg L^−1^ promoted reproduction and somatic growth.

**Figure 3:**
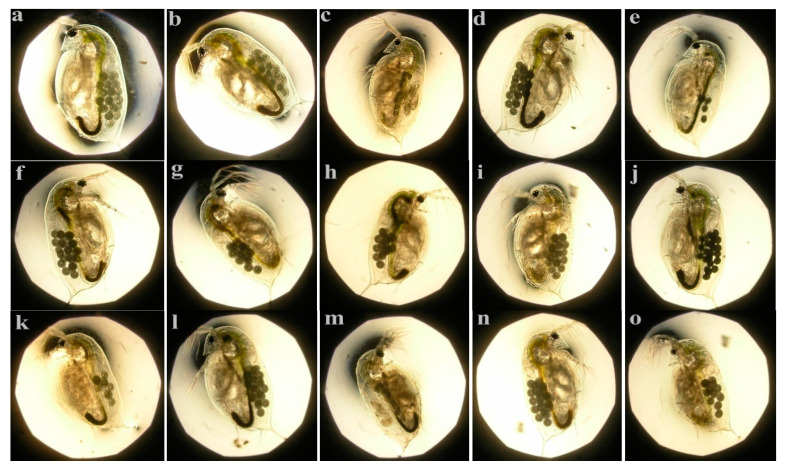
Microphotograph of offspring (9-d old) of *D. magna* **a** represents the control group; **b**, **d**, **f**, **h**, **j**, **l**, and **n** represent the treatment groups of DWTR, SNR-10, SR1-10, SR5-10, SNR-180, SR1-180, and SR5-180 at concentration of 5000 mg L^−1^; **c**, **e**, **g**, **I**, **k**, **m**, and **o** represent the treatment groups of DWTR, SNR-10, SR1-10, SR5-10, SNR-180, SR1-180, and SR5-180 at concentration of 500 mg L^−1^.

In addition, microphotographs of 9-day-old offspring of *D. magna* are shown in Figure 3. The body morphology of offspring had no difference among control, DWTR, and sediments amended with and without DWTR (at the incubation time of 10 and 180 d with 10% and 50% doses). Therefore, DWTR and DWTR-amended sediments did not have any effect on the embryonic development of *D. magna*.

### 3.5. Activities of Antioxidant Enzymes in D. magna

**Figure 4:**
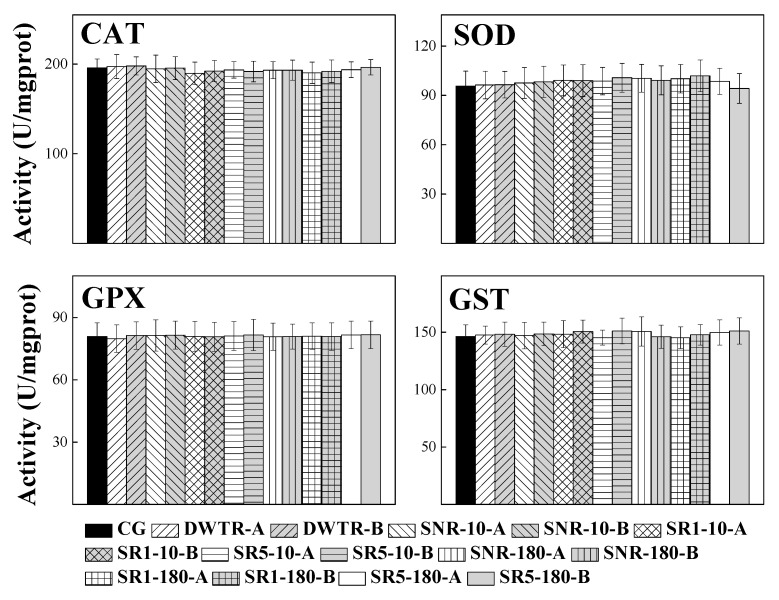
Antioxidant enzyme activities for catalase (CAT), superoxide dismutase (SOD), glutathione peroxidase (GPX), and glutathione-S-transferase (GST) in *D. magna*. A and B represent the concentrations of treatment culture at 5000 and 500 mg L^−1^, respectively; CG represents the control group.

The responses of antioxidant enzymes in *D. magna* are shown in Figure 4. The activities of CAT, SOD, GPX, and GST were similar in the control and the experimental treatments of DWTR and sediments amended with and without DWTR (at the incubation time of 10 and 180 d with 10% and 50% doses). Specifically, the activities of CAT, SOD, GPX, and GST were 189 (±12)–198 (±10), 94.2 (±9.1)–102 (±10), 79.8 (±6.6)–81.7 (±6.7) and 145 (±9)–151 (±11), respectively. Therefore, DWTR and DWTR-amended sediments had little effect on *D. magna* at the biochemical level.

## 4. Discussion

The bioassay was a useful way to measure the safety level of all contaminant components in pollutants [34]. The *D. magna* is commonly employed as a model organism given that it is a common member of aquatic invertebrate communities. Furthermore, *D. magna* is often considered a bioindicator species for studying environmental contamination because it has a short generation time, can be easily cultivated, and is sensitive to a wide range of contaminants [35,36,37]. Accordingly, determining the physiological and biochemical responses of *D. magna* to the dewatered DWTR could provide essential information about the safety of recycling DWTR in environmental remediation. The most important findings of this study were that the dewatered DWTR had no toxicity to *D. magna*, which was different from the results of previous studies, indicating the potential chronic toxic effect of the discharged DWTS on *D. magna* [24,25].

To determine the potential toxic effect of the dewatered DWTR on *D. magna*, the survival (Table 3), growth (Figure 1), life-history characteristics (Figure 2), body morphology of offspring (Figure 3), and antioxidant enzyme activities (Figure 4), which mainly reflected the physiological and biochemical characteristics of *D. magna*, were comprehensively examined in this study. Among these characteristics, previous studies have indicated that the abnormal body morphology of **D. magna** could be confirmed when the eyes were missing or malformed, the antennae were not fully developed, or the shell spine was not fully extended [32,38,39,40]. Furthermore, antioxidant enzyme activities, including CAT, SOD, GPX, and GST, have been proposed to provide early-warning indicators of the population-level effects of oxidative stress induced by exposure to sub-lethal concentrations [41]. Antioxidant defenses are often up-regulated by organisms to mitigate the excess production of reactive oxygen species (ROS) [42], as a large quantity of ROS induced by stressed conditions may cause oxidative stress and damage to cells [43]. The results of this study suggested that the survival, growth, reproduction, body morphology of offspring, and the antioxidant enzymes (CAT, SOD, GPX, and GST) of *D. magna* were not affected by the dewatered DWTR (Table 3 and Figure 1, Figure 2, and Figure 4), indicating the safety of the dewatered DWTR to *D. magna*.

The potential chronic toxic effect of the discharged DWTS on *D. magna* was mainly caused by the heavy metals contained, especially Zn, Pb, Ni, Cu, and Cr [24,25]. To explore the mechanisms for the contrary observation of the toxic effect between the dewatered DWTR and the discharged DWTS, the distributions of these metals in the dewatered DWTR were investigated based on total contents analysis and the BCR method. Sequential extraction (e.g., BCR method) applied progressively more destructive reagents to provide insight into the bound of trace elements to solid phases [44]. For metals extracted using the BCR method, the acid-soluble fraction has the highest mobility and is commonly considered to have high pollution risks, which were followed by reducible, oxidizable, and residual fractions, respectively [27]. Accordingly, a further comparison suggested that the total contents of Pb, Ni, and Cu in dewatered DWTR (Table 2) were substantially lower than those in the discharged DWTS reported in a previous study (Table S1) [24]. The relatively higher concentrations of Zn and Cr were observed in the dewatered DWTR compared to DWTS (Table S1), but Zn and Cr, as well as Pb, Ni, and Cu, in DWTR were not mainly distributed in the mobile fraction, especially Pb, Cu, and Cr, which was dominated by oxidizable and/or residual fractions (Table 2). These findings demonstrated that the different toxicity between the dewatered DWTR and the discharged DWTS could be due to the varied heavy metal pollution risk. Heavy metals with relatively low concentrations and high stability contributed to the high safety of the dewatered DWTR.

The reasons for relatively low heavy-metal pollution risks of the dewatered DWTR could be explained by the following two aspects. On the one hand, the relatively low heavy-metal content in the dewatered DWTR may be related to the dewatering treatment (to DWTS). The potentially toxic part of heavy metals in the discharged DWTS were contributed mainly from flocculants utilization [24,25]. Given that the flocculation treatment was a conventional technique in different waterworks [1], the potential high pollution risks of heavy metals on *D. magna* could be the general character of the discharged DWTS from different waterworks. Such information indicated that the dewatering treatment may remove some heavy metals from DWTS. However, additional work is needed to confirm this hypothesized consequence of dewatering treatment on DWTR toxicity.

On the other hand, the relatively high stability of heavy metals in dewatered DWTR could be related to the treatment of air-drying to fresh dewatered DWTR, which has been demonstrated in a previous study [45]. The fresh dewatered DWTR was often block shaped in irregular form and with relatively high moisture contents. The sequential treatments of air-drying and crushing to the dewatered DWTR (as the preparation used in this study) were often adopted, because the treatments were beneficial for samples to be transported and stored, promoting the recycling of DWTR. This study further demonstrated that the air-drying was a necessary pretreatment method to enhance the safety of the dewatered DWTR for recycling. In addition, Al may be toxic to organisms under certain conditions (e.g., at pH < 3) [46], but no toxic effect on *D. magna* was observed herein. This phenomenon was caused by the fact that DWTR showed an alkalescent effect and Al in DWTR was dominant in oxidizable and residual fractions with high stability (Table 2), although Al in DWTR was at relatively high concentrations.

Another interesting finding of this study was that sediment (without DWTR) had no undesirable effect on the *D. magna* (Table 3 and Figure 1, Figure 2, and Figure 4); even, sediments at the concentration of 5000 mg L^−1^ had a positive effect on *D. magna*, which reflected in promoting the reproduction and growth of *D. magna* (Figure 1 and Figure 2). The promotion could be because sediments at high concentrations supplied abundant nutrient substances for the animals. Previous studies have reported that natural particles at relatively high concentrations (>200 mg L^−1^) were beneficial for the health of *D. magna* [47], and that *D. magna* could ingest solid particles with diameters less than 40 μm as food [48]. This study found that 38.2–41.4% of solid particles in sediments (without DWTR) had diameters less than 40 μm, indicating the potential utilization of sediments for survival by *D. magna*. Notably, DWTR at different concentrations did not exhibit any promotion on the survival of *D. magna*, which may be due to the limitation of solid particles with diameters less than 40 μm (2.45%) in DWTR that could be provided for *D. magna*.

However, the positive effect of sediments on *D. magna* was maintained for sediment after being amended by DWTR at different incubation times and doses (Figure 1 and Figure 2). The positive effect could also be due to the supplement of nutrients for *D. magna* by the DWTR amended sediments, because 27.3–37.1% of solid particles were found with diameters less than 40 μm in the amended sediment. Furthermore, the positive effect could be supported by the observed limited variations of heavy metals pollution risks of sediments after being amended by DWTR. The addition of DWTR had a limited effect on total heavy metals contents (Pb, Ni, Cu, Cr, and Zn) in sediment. The addition mainly induced the transformations of heavy metals between oxidizable and residual fractions, with a minor effect on metals mobility in sediments, although the distributions of heavy metals in sediment and DWTR had some differences. Examining the biological safety of DWTR should be based on assessing the toxicity to organisms at different trophic levels [49,50]. It has been reported that the dewatered DWTR had no toxic effect on the producer *C. vulgaris* [22] and the decomposer *A. fischeri* [21]. Contrasting to the trophic levels of the producer and decomposer, *D. magna* tested herein can represent the consumer in an aquatic environment. According to the toxicity tests of this study and previous studies, the dewatered DWTR could be safe for environmental remediation.

## 5. Conclusions

This study comprehensively assessed the effects of DWTR and lake sediments amended with and without DWTR (at different doses and incubation times) on the survival, growth, life-history traits, embryonic development, and antioxidant enzyme activities of *D. magna*. The results showed that DWTR and sediments with and without DWTR did not inhibit the survival, growth, and reproduction of *D. magna*. The rate of increase in body weight and length, age at first pregnancy and first reproduction, average number of offspring, and length at 21 d were even promoted when *D. magna* was exposed to 5000 mg L^−1^ sediments with and without DWTR. This enhancement may stem from the extra nutrition supplied by sediments with and without DWTR at 5000 mg L^−1^ for the animals. All offspring of *D. magna* in DWTR and sediments with and without DWTR had bodies with normal morphologies just like control animals. The activities of antioxidant enzymes, including CAT, SOD, GPX, and GST, in *D. magna* were similar in the control and the experimental treatments. These results indicated that DWTR had no adverse effects on *D. magna*, and the addition of DWTR had limited impact on the effect of sediments on *D. magna*. Further analysis suggested that heavy metals with relatively low concentrations and high stability contributed to the high safety of the dewatered DWTR. Overall, DWTR can be considered a non-hazardous material for zooplankton.

## Figures and Tables

**Table 1:** The scheme of biological tests.

Test	Content	Incubation Condition	Index
Bioassay 1	The survival of *D. magna*	Incubation time: 48 h; sample concentrations: 0–5000 mg L^−1^; conditions: 25 ± 0.5 °C with a 16–8 h light-dark photoperiod	Mortality
Bioassay 2	The growth of *D. magna*	Incubation time: 5 d; sample concentrations: 0–5000 mg L^−1^; conditions: 25 ± 0.5 °C with a 16–9 h light-dark photoperiod	Body length and weight
Bioassay 3	The life-history traits of *D. magna*	Incubation time: 21 and 9 d; sample concentrations: 0–5000 mg L^−1^; conditions: 25 ± 0.5 °C with a 16–10 h light-dark photoperiod	First pregnancy, time to release the first brood, neonates numbers, body lengths, and microphotographs
Bioassay 4	The antioxidant enzymes of *D. magna*	Incubation time: 48 h; sample concentrations: 0–5000 mg L^−1^; conditions: 25 ± 0.5 °C in darkness	CAT, SOD, GPX, and GST

**Table 2:** The properties of drinking water treatment residue (DWTR) and sediments.

Parameter		Dewatered DWTR	Raw Sediment	SNR-10	SNR-180	SR1-10	SR1-180	SR5-10	SR5-180
pH		7.34 ± 0.54 ^1^	7.82 ± 0.61	7.84 ± 0.62	7.81 ± 0.57	7.78 ± 0.47	7.75 ± 0.68	7.65 ± 0.61	7.67 ± 0.64
Total organic matter (mg g^−1^)		85.3 ± 5.67	67.8 ± 5.24	69.2 ± 5.35	66.9 ± 4.95	69.6 ± 4.68	71.3 ± 5.07	77.5 ± 5.2	79.1 ± 5.83
Fe	Totalcontent (mg g^−1^)	130 ± 9.90	35.2 ± 2.16	37.2 ± 2.69	33.8 ± 2.04	46.7 ± 3.37	48.4 ± 3.27	78.6 ± 5.82	84.0 ± 6.27
	Acid-soluble ^2^	0.501%	0.281%	0.523%	0.187%	0.271%	0.192%	0.231%	0.210%
	Reducible ^2^	13.3%	7.65%	5.54%	8.53%	6.93%	5.85%	7.17%	6.29%
	Oxidizable ^2^	58.7%	2.26%	4.44%	2.09%	10.4%	9.96%	27.3%	25.2%
	Residual ^2^	27.5%	89.8%	89.5%	89.2%	82.4%	84.0%	65.3%	68.3%
Al	Total content (mg g^−1^)	98 ± 6.78	61.3 ± 4.04	57.5 ± 5.10	60.6 ± 4.42	68.7 ± 4.95	63.1 ± 5.02	74.4 ± 5.25	80.3 ± 6.21
	Acid-soluble	11.5%	0.0176%	0.0239%	0.117%	0.0193%	0.0147%	0.114%	0.109%
	Reducible	4.03%	0.123%	0.117%	0.105%	0.181%	0.186%	0.286%	0.291%
	Oxidizable	56.2%	2.56%	1.86%	1.48%	16.9%	15.5%	32.7%	31.1%
	Residual	28.3%	97.3%	98.0%	98.3%	82.9%	84.3%	66.9%	68.5%
Zn	Total content (mg g^−1^)	0.0643 ± 0.00512	0.0700 ± 0.0100	0.0721 ± 0.0100	0.0689 ± 0.00923	0.0631 ± 0.00541	0.0619 ± 0.00420	0.0632 ± 0.00210	0.0647 ± 0.00516
	Acid-soluble	35.2%	5.35%	4.22%	4.53%	5.23%	8.27%	9.07%	6.19%
	Reducible	27.6%	6.73%	5.78%	5.52%	5.67%	5.03%	6.23%	5.11%
	Oxidizable	25.3%	7.35%	8.1%	7.15%	10.2%	9.10%	14.1%	13.6%
	Residual	11.9%	80.6%	81.9%	82.8%	78.9%	77.6%	70.6%	75.1%
Pb	Total content (mg g^−1^)	0.0146 ± 0.00111	0.0110 ± 0.0120	0.0131 ± 0.00268	0.0124 ± 0.00109	0.0161 ± 0.00131	0.0138 ± 0.00111	0.0101 ± 0.00161	0.0162 ± 0.00113
	Acid-soluble	ND ^3^	ND	ND	ND	ND	ND	ND	ND
	Reducible	4.43%	1.12%	1.92%	0.910%	2.52%	1.51%	0.710%	1.20%
	Oxidizable	55.5%	38.5%	37.8%	35.3%	18.1%	15.6%	16.2%	17.1%
	Residual	40.1%	60.4%	60.3%	63.8%	79.4%	82.9%	83.1%	81.7%
Ni	Total content (mg g^−1^)	0.0147 ± 0.00184	0.0110 ± 0.00114	0.0124 ± 0.00192	0.0113 ± 0.00211	0.0137 ± 0.000961	0.0146 ± 0.00107	0.0124 ± 0.00152	0.0137 ± 0.00184
	Acid-soluble	15.9%	3.84%	2.51%	5.21%	2.48%	4.87%	5.14%	3.87%
	Reducible	20.5%	1.13%	1.76%	1.06%	1.68	0.960%	2.03%	2.62%
	Oxidizable	32.8%	6.43%	6.83%	4.13%	6.54%	4.57%	8.23%	7.01%
	Residual	30.8%	88.6%	88.9%	89.6%	89.3%	89.6%	84.6%	86.5%
Cu	Total content (mg g^−1^)	0.0300 ± 0.00225	0.0281 ± 0.00304	0.0289 ± 0.00314	0.0235 ± 0.00217	0.0302 ± 0.00216	0.0314 ± 0.00215	0.0293 ± 0.00101	0.0286 ± 0.00123
	Acid-soluble	2.1%	0.121%	0.184%	0.121%	0.139%	0.147%	0.233%	0.193%
	Reducible	0.601%	0.239%	0.176%	0.239%	0.161%	0.153%	0.167%	0.507%
	Oxidizable	48.2%	7.54%	5.24%	7.54%	10.6%	10.0%	27.4%	25.2%
	Residual	49.1%	92.1%	94.4%	92.1%	89.1%	89.7%	72.2%	74.1%
Cr	Total content (mg g^−1^)	0.0838 ± 0.00604	0.0570 ± 0.00456	0.0590 ± 0.00413	0.0619 ± 0.00412	0.0631 ± 0.00548	0.0602 ± 0.00712	0.0744 ± 0.00525	0.0629 ± 0.00323
	Acid-soluble	4.16%	0.151%	0.133%	0.227%	0.192%	0.232%	0.119%	0.159%
	Reducible	2.83%	0.239%	0.137%	0.103%	0.228%	0.108%	0.261%	0.121%
	Oxidizable	7.91%	3.31%	3.23%	3.27%	3.88%	4.06%	5.42%	3.22%
	Residual	85.1%	96.3%	96.5%	96.4%	95.7%	95.6%	94.2%	96.5%
Particle percentiles ^4^ (%)		2.45 ± 0.169	39.8 ± 3.14	38.2 ± 3.05	41.4 ± 3.31	37.0 ± 2.69	37.1 ± 2.48	27.3 ± 2.14	30.1 ± 2.08

^1^ Mean ± standard deviation (SD, n = 3). ^2^ Acid-soluble, Reducible, Oxidizable, and Residual represent acid-soluble, reducible, oxidizable, and residual fractions of metals; the residual fraction was calculated based on subtracting acid-soluble, reducible, and oxidizable fractions from total metal. ^3^ Not detectable. ^4^ The percentiles (%) of solid particles with a diameter less than 40 μm.

**Table 3:** Survival rate (%) of *D. magna* exposed to DWTR and sediments amended with and without DWTR in an acute test.

Samples	Concentration (mg L^−1^)					
	0	5	50	100	500	5000
DWTR	96	100	100	96	100	100
SNR-10	96	98	100	100	100	100
SR1-10	96	100	96	100	100	100
SR5-10	96	100	100	100	100	100
SNR-180	96	100	100	98	100	100
SR1-180	96	100	100	100	100	100
SR5-180	96	100	100	100	100	100

## Supplementary Materials

The following are available online at https://www.mdpi.com/1660-4601/17/16/5863/s1: S1: The detailed biological experiments, Figure S1: Sensitivity estimation (LC50, 48-h) of *D. magna* to K2Cr2O7, Table S1: The total metal contents (mg kg^−1^) of drinking water treatment sludge (DWTS).
