# Supplementary material for: The Physiological and Biochemical Responses of Daphnia magna to Dewatered Drinking Water Treatment Residue
Source: Int J Environ Res Public Health. 2020 Aug 13;17(16):5863. doi: 10.3390/ijerph17165863 (PMC7460191; doi:10.3390/ijerph17165863)
Supplement: Supplementary file 1 [file ijerph-17-05863-s001.pdf]

Supporting information for

The physiological and biochemical responses of *Daphnia magna* to dewatered drinking water treatment residue

Nannan Yuan[1,2,3], Yuansheng Pei[3], Anping Bao[1], Changhui Wang[2,*]

[1] *Electronic Information Technology School, Nanjing Vocational College of Information Technology, Nanjing 210023, China.*

[2] *State Key Laboratory of Lake Science and Environment, Nanjing Institute of Geography and Limnology, Chinese Academy of Sciences, Nanjing 210008, China.*

[3] *State Key Laboratory of Water Environment Simulation, Key Laboratory for Water and Sediment Sciences of Ministry of Education, School of Environment, Beijing Normal University, Beijing 100875, China.*

[*]Corresponding Author: Tel.: 86-025-86882210; *E-mail address*: chwang@niglas.ac.cn (C.H. Wang)

S1. The detailed biological experiments

Four sets of tests were performed for all seven samples (DWTR, SNR-10, SR1-10, SR5-10, SNR-180, SR1-180, and SR5-180). The test media with different sample concentrations were prepared by diluting stock suspensions with STM culture media. Media were made the day before each renewal to obviate the potential effects of suspended particles. Neonates less than 24 h in age were used for the first three sets of experiments (i.e., bioassay 1-3), and *D. magna* at least 5 days in age were used for the last sets of tests (i.e., bioassay 4). Bioassay 1, 3 and 4 were conducted under the same temperature and light conditions as described in section 2.3, and bioassay 2 was conducted at 25 ± 0.5 °C in darkness. The detailed descriptions of bioassay 1-4 were shown below.

Bioassay 1 was conducted under static, non-renewal conditions for 48 h to determine the mortality of *D. magna* in 50 mL glass beakers with 20 mL of test media. Samples were tested at 0, 5, 50, 100, 500, and 5000 mg L^{-1}. Five replicates with five *D. magna* in each repeat were conducted. Before the test, the organisms were fed *Scenedesmus* for 2 h.

Bioassay 2 was performed to determine the growth of *D. magna* with 100 mL of test media at sample concentrations of 0, 5, 50, 100, 500, and 5000 mg L^{-1}. Five replicates with five *D. magna* in each repeat were conducted. Neonates in each beaker were fed with *Scenedesmus* at a concentration of 5 mg-C L^{-1}. At the beginning of the tests, the body lengths of 15 neonates were measured and five groups of 5 neonates were dried at 105 °C for 24 h and then weighed [41]. After incubation for 5 days, experimental *D. magna* were collected and rinsed in STM medium, and then the body lengths and dry weights were measured. Growth rates were determined as the increase in dry mass and body length from the beginning of the experiment (W_0 and L_0) to day 5 (W_5 and L_5) using the following equations:

$$\text{Body weight growth rate} = (\ln W_5 - \ln W_0) / 5 \quad (1)$$

$$\text{Body length growth rate} = (\ln L_5 - \ln L_0) / 5 \quad (2)$$

Bioassay 3 was conducted to examine the responses of life-history traits of *D. magna* at sample concentrations of 0, 500, and 5000 mg L^{-1}. *Scenedesmus* was added daily to all

beakers as food. Test media were renewed every second day. Ten replicates with only one *D. magna* in each repeat were conducted. The numbers of survivors, time to first pregnancy, and time to release the first brood were recorded, and newborns were removed and counted to obtain the average number of neonates produced by each organism during the 21-day test period. At the end of the exposure period, the body lengths of animals were measured. Offspring born during the first brood of test organisms was transferred to and cultured in fresh test medium for 9 days under the same temperature, light, and food conditions. Next, offspring were inspected and photographed under a binocular microscope and classified either as normally or abnormally developed.

Bioassay 4 was conducted to analyze the antioxidant enzymes of *D. magna* after 48 h of exposure to samples at concentrations of 0, 500, and 5000 mg L^{-1}. Five replicates of five *D. magna* each were conducted. The tests were performed under static non-renewal conditions in 50 mL of test solution without feeding. The organisms were then transferred to a 2 mL centrifuge tube containing 1.5 mL of 0.05 M Tris-HCl buffer with a pH of 8.2 and were grounded with an ultrasonic tissue destructor (Sonics Uibra Cell VCX 105, Sonics & Materials Inc., USA). All procedures were completed on ice. The homogenates were then centrifuged at 10000 rpm for 20 min at 4 °C, and the supernatant was used for analyses of antioxidant enzymes. The activities of catalase (CAT), superoxide dismutase (SOD), glutathione peroxidase (GPX), and glutathione-S-transferase (GST) were determined using the reagent kits (Nanjing Jiancheng Bioengineering Institute) per the manufacturer's instructions.

Table S1. The total metal contents (mg kg^{-1}) of drinking water treatment sludge (DWTS).

Metals	$FeCl_3$ sludge[a]	
	Wet season	Dry season
Zn	31.5	39.5
Pb	48.0	46.0
Ni	78.0	50.0
Cu	34.0	57.0
Cr	42.0	34.0

[a] The total metal contents were referred to Sotero-Santos et al. (2005).

Sotero-Santos RB, Rocha O, Povinelli J. Evaluation of water treatment sludges toxicity using the *Daphnia* bioassay. Water Res. 2005;39 (16):3909-3917.

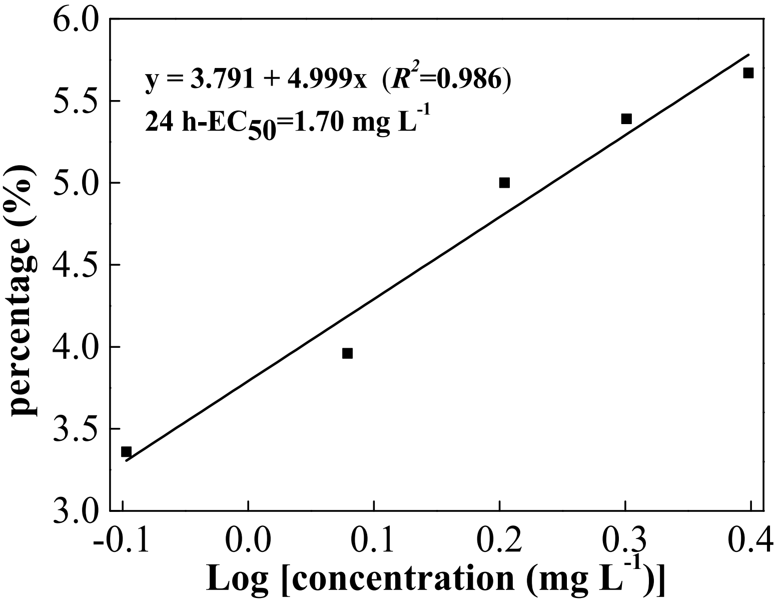

Figure S1. Sensitivity estimation (LC50, 48-h) of *D. magna* to $K_2Cr_2O_7$.
